# Horizontal-vertical movement relationships: Adélie penguins forage continuously throughout provisioning trips

**DOI:** 10.1186/s40462-021-00280-8

**Published:** 2021-08-26

**Authors:** Javed Riaz, Sophie Bestley, Simon Wotherspoon, Louise Emmerson

**Affiliations:** 1 Institute for Marine and Antarctic Studies, University of Tasmania, Private Bag 129, Hobart, TAS 7001 Australia; 2 Australian Antarctic Division, 203 Channel Highway, Kingston, TAS 7050 Australia

**Keywords:** Foraging behaviour, *Pygoscelis adeliae*, Area-restricted search, Horizontal movement, Dive behaviour, Habitat use

## Abstract

**Background:**

Diving marine predators forage in a three-dimensional environment, adjusting their horizontal and vertical movement behaviour in response to environmental conditions and the spatial distribution of prey. Expectations regarding horizontal-vertical movements are derived from optimal foraging theories, however, inconsistent empirical findings across a range of taxa suggests these behavioural assumptions are not universally applicable.

**Methods:**

Here, we examined how changes in horizontal movement trajectories corresponded with diving behaviour and marine environmental conditions for a ubiquitous Southern Ocean predator, the Adélie penguin. Integrating extensive telemetry-based movement and environmental datasets for chick-rearing Adélie penguins at Béchervaise Island, we tested the relationships between horizontal move persistence (continuous scale indicating low [‘resident’] to high [‘directed’] movement autocorrelation), vertical dive effort and environmental variables.

**Results:**

Penguins dived continuously over the course of their foraging trips and lower horizontal move persistence corresponded with less intense foraging activity, likely indicative of resting behaviour. This challenges the traditional interpretation of horizontal-vertical movement relationships based on optimal foraging models, which assumes increased residency within an area translates to increased foraging activity. Movement was also influenced by different environmental conditions during the two stages of chick-rearing: guard and crèche. These differences highlight the strong seasonality of foraging habitat for chick-rearing Adélie penguins at Béchervaise Island.

**Conclusions:**

Our findings advance our understanding of the foraging behaviour for this marine predator and demonstrates the importance of integrating spatial location and behavioural data before inferring habitat use.

**Supplementary Information:**

The online version contains supplementary material available at 10.1186/s40462-021-00280-8.

## Background

All animals must forage and acquire energy to survive and maximise fitness. An individual’s foraging behaviour and strategies can have significant implications for foraging and breeding success, and ultimately drive population-level trends and characteristics (e.g., distribution, size, density, mortality, fecundity, health, offspring quality) [1–3]. Therefore, understanding foraging movements and habitat use is critical for ecosystem conservation planning and resource management efforts, and has been a long-standing objective in ecological research [4, 5]. Air-breathing marine predators, such as seabirds and marine mammals, present a special case as they forage in a dynamic fluid environment, for prey distributed throughout the horizontal and vertical planes, and must do so within the limits imposed by oxygen stores used while diving [6, 7].

For diving marine predators, bio-logging and telemetry devices remain the most practical means of inferring foraging effort and behaviour over the longer term [8]. Although ideal to understand how predators interact with their prey field, direct observations (e.g., via *in-situ* video footage and stomach temperature sensors) of underwater foraging behaviour are difficult to obtain and typically provide only short-term information [9–11]. To provide a more extensive dataset of foraging movements of marine animals at sea, a combination of two-dimensional horizontal movement track and a one-dimensional trace of diving activity through time are more commonly recorded.

Horizontal and vertical movements are generally recorded at different spatiotemporal resolutions. Consequently, studies which seek to quantify marine predator foraging behaviour have traditionally considered only one aspect of movement; examining either horizontal or vertical movement separately and using optimal foraging theory (OFT) to make foraging inferences [12, 13]. Originating within terrestrial ecology, OFT originally tested mainly two-dimensional horizontal movement models, but this conceptual framework is widely used to examine the mechanisms and strategies an animal uses to acquire food across aquatic, terrestrial and aerial species. According to OFT, animals should maximise the time spent in areas of higher energetic gain and profitability, while minimising energetic costs associated with prey acquisition [3]. Horizontal movement studies are traditionally based around the concept of area-restricted search (ARS) behaviours, where animals are expected to concentrate foraging efforts in areas of high prey density [14]. Animal trajectories will therefore be expected to switch between ‘resident’ movement within prey patches, and more ‘directed’ movement patterns between prey areas [15]. Directed movements are characterised by high speed and linear directionality, while resident movement represents reduced displacements and increased turning rates (putatively labelled ‘searching’ or ‘foraging’ behaviour) [6, 16, 17]. For diving marine predators, movement in the vertical dimension is also expected to correspond to similar movement patterns. While in areas of high prey density, marine predators are predicted to optimise time allocation within a dive, maximising time spent at foraging depths, energy efficiency and prey capture attempts, and minimizing transit time to and from the forage areas at depth [13, 18, 19].

Recent explorations of horizontal-vertical movement relationships through dual-tagging efforts (i.e. simultaneously deploying satellite telemetry and dive logger devices) have challenged these long-standing optimal foraging assumptions. While some studies have validated the correspondence between ARS and pronounced diving effort [12, 20, 21], others have found a spatial and temporal disconnect between the two and support the idea that frequently applied movement models may oversimplify complex behaviours [6, 22, 23]. Variability in horizontal-vertical movement relationships, even within the same species [e.g., southern elephant seals; 24, 25], challenge the universal applicability of simplified OFT to all marine predator taxa.

Generally, efforts to link movement in the horizontal and vertical dimension adopt a multi-step approach. This involves: (1) filtering horizontal tracks to account for location errors; (2) ascribing foraging components using geometry of movement trajectories [e.g., phenomenological approaches like first passage time; [15] or process-based models ([e.g., hidden Markov models; 26, 27, 28]); and (3) statistically linking horizontal and vertical movements [29]. However, the complex nature of telemetry-based animal movement data are driving increasingly sophisticated analytical efforts to integrate these steps [16, 30–32].

State-space models (SSMs) are process-based models which have emerged as a valuable tool to explore complex movement behaviours [31, 33]. Movement processes are parameterised within a state-switching framework, where animals switch between discrete movement states. This commonly involves a two state-switching model between foraging and transit behavioural states, but can also been expanded to include three or more behaviours (e.g. resting, exploring or predator evasion) [27, 30]. These discrete frameworks can also account for observation errors [6, 34]. More realistically, animal movement likely occurs over a dynamic behavioural continuum [35]. Models which use time-varying parameters to examine movement as a continuous behavioural index are relatively less common; however, they can provide a more nuanced insight into changes in movement behaviour and yield a greater biological realism [35–37]. When integrated with ancillary information, such as diving and environmental data, such movement models can improve understanding of foraging strategies and habitat use in the different dimensions [36, 37].

Adélie penguins are one of the most studied polar seabirds on the planet. As Antarctic predators regarded as ecosystem indicators, they are a key species within the Commission for the Conservation of Antarctic Marine Living Resources (CCAMLR) Ecosystem Monitoring Program (CEMP) [38], and their foraging behaviour has been extensively studied. Numerous studies have investigated Adélie penguin movement, focussing on either the horizontal [39–41] or vertical [42–45] dimension. However, few studies have correlated movement in the two domains to provide a more holistic understanding of foraging behaviour [46–51]. This represents a critical gap in our understanding of Adélie penguin habitat use, and in particular, the precise details of where they are capturing prey. Such integrated information can improve the utility of marine predators as indicator species for assessing the state of marine resources, and informing ecosystem-level spatial conservation and fisheries management [52, 53].

In this study, we investigate how changes in movement behaviour are related in the horizontal and vertical dimensions for chick-rearing Adélie penguins from the Béchervaise Island colony in East Antarctica. We also examine how movement relates to key environmental features to identify important foraging habitats during guard and crèche stages of the chick-rearing period. We utilise archived multi-year telemetry datasets from birds which were dual-tagged with platform terminal transmitters (PTTs) and time-depth recorders (TDRs), providing both spatial location and dive information. Adopting a time-varying approach [36], we estimate movement persistence along the trajectories and examine how change in horizontal movement corresponds with vertical foraging effort (summed diving activity) and environmental variables. We tested whether (1) foraging dive effort increased during times of more resident behaviour along movement trajectories, thereby validating single-dimension approaches to examining foraging behaviour, and (2) horizontal-vertical foraging movements were influenced by different environmental conditions during guard and crèche. By directly integrating horizontal movements with diving and environmental information, we provide an improved understanding of Adélie penguin foraging strategies and spatiotemporal patterns of forage resources used by this colony.

## Methods

### Data collection

Béchervaise Island in East Antarctica (67°35 S, 67°49 E), is an Adélie penguin nesting site which is home to over 2000 breeding pairs [54]. It has been a designated CEMP site since 1990, and the focus of a long-term Adélie penguin monitoring program. Platform terminal transmitters (PTTs) and time-depth recorders (TDRs) were deployed on foraging Adélie penguins at Béchervaise Island over the breeding seasons between 1994/1995 and 2003/2004. To integrate movements in the horizontal and vertical dimension and test optimal foraging assumptions, we collated telemetry data from 6 breeding seasons (excluding 1992/1993, 1997/1998 and 1999/2000, where data were either not retrieved or useable due to device malfunction). This dataset comprised of 23 dual-tagged individuals (13 females, 10 males) over 27 foraging trips during the chick-rearing period: guard (late-December to mid-late January) through crèche (mid-January to early-mid-February) (Additional file 1: Table S1).

During the chick-rearing stages, foraging movements are constrained by different intrinsic and extrinsic factors. At Béchervaise Island, guard is characterised by short (< 60 km) and alternating foraging trips between breeding pairs, generally, within an environment composed of extensive fast ice. In crèche, chicks become thermally independent, and parents must forage simultaneously to acquire enough food to provision chicks. Parents perform longer foraging trips (< 125 km), which generally coincides with the reduction of sea ice adjacent the breeding colony and greater access to more distant prey-rich locations [39, 40, 55].

During the study period, PTTs of three different makes and models were deployed. Individuals were captured at nests and the status of chicks as either in guard or crèche stage were made. Sex was also determined by cloacal examination [56]. All PTT devices were glued to feathers on the lower back using rapid-hardening epoxy glue (Loctite 401™) and secured with cable ties threaded under the feathers and around the device. The devices were shaped to minimise hydrodynamic drag and were packaged by Sirtrack to withstand diving to 200 m [39]. In the years between 1994 and 1999, Toyocom T-2038 (100 g) and Telonics ST-6 and ST-10 (120 g) were used. The weight of these loggers was approximately 2.3% and 2.7% of the penguins’ body mass, respectively. The frontal cross-sectional areas of these devices were approximately 7cm^2^. This represented 1.7% of the penguins’ frontal cross-sectional area. From the year 2000, Kiwisat 101 (Sirtrack) (90 g) PTT devices, which had a cross-sectional area of 3.75 cm^2^ were deployed. The deployment and programming of PTT devices are described in full in Clarke et al. [39]. An Automated Penguin Monitoring System (APMS) was used to monitor the time of departure and arrival of individually tagged penguins from the colony, enabling foraging trip duration records [57].

Over the same 6 breeding seasons, dive records of chick-rearing Adélie penguins were also obtained using TDR devices. Before 1999, Wildlife Computers Mk5 TDRs (Redmond, USA; 50 g; 65 × 35 × 15 mm) were used, which recorded depth in 5, 2 or 1 s increments with a ± 1 m resolution. From the year 2000 onwards, Mk7 TDRs (30 g; 98.5 × 20 × 10 mm), recording depth every 1 s with a ± 0.5 m resolution, were deployed. These loggers were approximately 0.7% and 1% of the penguin’s body mass, respectively. Full details of TDR deployments and dive data processing are provided in Riaz et al. [43].

### Dive analyses

Archived dive data were downloaded using Wildlife Computers software packages and a zero-offset correction applied to depth profiles. To account for surface noise, we excluded dives < 3 m from our analyses [43]. All subsequent data processing and analyses were performed using R statistical software version 3.5.1 [58]. For each dive, we identified: (1) maximum depth (m); (2) dive duration (s); (3) surface interval (s); (4) bottom duration (s), defined as the amount of time spent within 50% of the maximum dive depth where the rate of change in depth during descent or ascent did not exceed 50%; (5) and wiggles, comprising the number of undulations in the dive profile > 2 m in depth (ascent to descent).

### Spatial location quality control and filtering

Raw PTT tracks of 23 chick-rearing penguins with coinciding TDR data were assembled (n = 5393 ARGOS locations), plotted and visually inspected. The periods at the start and end of each foraging trip were removed where it was clear location fixes were onshore, and therefore not representative of at-sea movement (n = 928). Locations were then subjected to automated quality-control checks largely based on the data filtering processes used in Ropert-Coudert et al. [59]: (1) near-duplicate location estimates occurring within 120 s of each other were removed (n = 322); (2) any Z-class locations were reclassified as B-class locations (n = 10); (3) location estimates with travel rates exceeding 10 m s^−1^ were removed (n = 15) (4) foraging trips which were comprised of fewer than 10 location estimates were removed (n = 0); and (5) foraging trips durations less than 1 day were removed (n = 0) (Table 1).

**Table 1:** Count of raw ARGOS location fixes available at each step in the data quality-control procedure, and the location estimates available following SSM filtering and integration of horizontal-vertical data streams

Step-wise quality controls	N fixes
Raw tracks	5393
Onshore locations removed	4465
Near-duplicates removed (< 120 s)	4143
Unrealistic travel rates (< 10 m/s) removed	4143
Trips < 10 locations removed	4128
Trips < 1 day removed	4128

SSM processing	N estimates
Regularised (1 h) tracks	3256
Location estimates between first and last dive	2886
Corresponding PTT and TDR data	2220

Data represent 23 breeding individuals over 27 foraging trips

We fitted a continuous-time correlated random walk SSM to the quality-controlled PTT locations using the *‘fit_ssm’* function in the ‘*foieGras’* package [60]. See Jonsen et al. [61] for process model equations. This approach accounted for observation errors in tracking data, and also provided location estimates and standard errors at regular time steps along the track [31, 59]. Autocorrelation in successive displacements is sensitive to the time-steps used to define those displacements [61]. Therefore, we first tested various time-steps (1, 2 and 3 h) and compared move persistence parameters against continuous-time results fitted to irregular location estimates (i.e., the *‘predicted’* against the ‘*fitted’* values generated by the *‘fit_ssm’* function to assess predictive performance) (Fig. S1). Spatial location estimates at regular 1 h time intervals were identified as being most practical and adequate for our purposes (n = 3256; Table 1) in linking the horizontal and vertical data streams.

### Horizontal-vertical data integration

The dive data for the 23 individuals were collated from Riaz et al. [43] and binned into 1 h time periods corresponding to SSM location estimates. To examine underwater behaviour and quantify total foraging dive effort, we calculated the sum of the maximum depth (m), dive bottom duration (s), and number of wiggles of all dives performed every hour for the duration of the foraging trip. Summing dive parameters in this way is commonly used to quantify the foraging effort and total vertical movement of penguins throughout the water column [62, 63]. We also calculated attempts of catch per unit effort [ACPUE; [64]; Eq. 1].

$$\mathrm{ACPUE}= \frac{\mathrm{Total\, number\, of\, wiggles}}{\mathrm{Total\, time\,in\,bottom\,duration}}$$

At Béchervaise Island, chick-rearing Adélie penguins often walk or toboggan across nearshore fast ice adjacent the breeding colony to access foraging grounds [40]. To account for these non-aquatic components of trajectories, we excluded location estimates before the first dive and after the last dive (n = 370; Table 1). On average, excluded location estimates accounted for 1.55% (95% CI: 0.1 to 28.8%) of foraging trip durations. To ensure we were not removing shallow aquatic transiting behaviour < 3 m, we examined the speed of excluded movement trajectories. Adélie penguins are known to walk over fast ice at approximately 2 km h^−1^. The mean speed of our excluded movement components was 1.44 km h^−1^ ± 1.45 km h^−1^, giving us confidence in our non-aquatic movement designation. The horizontal-vertical analysis incorporated only location estimates during the foraging trip where corresponding diving information was available, so the final dataset represents 77% of the 1 h timesteps identified as aquatic (n = 2220).

### Environmental data

To examine how Adélie penguin movement behaviour varied in relation to environmental conditions, we extracted a suite of environmental variables along penguin tracks using the *raadtools* package [65]. A range of static and dynamic environmental variables known to influence penguin movement behaviour were extracted at each individual state-space filtered location estimate [52]. This included bathymetry (BATH), bathymetry slope (BS), sea-ice concentration (SIC), sea surface temperature (SST) and sea surface height (SSH) (Table 2). These data were appended to our integrated horizontal-vertical movement record, providing an environmental context for movement behaviour.

**Table 2:** Static and dynamic environmental predictors used to examine Adélie penguin movement-environment relationships

Covariate type	Predictor	Description
Static variables	Bathymetry (BATH)	Estimated sea floor depth (m) at a 0.02° × 0.02° spatial resolution [66]. Influences the horizontal and vertical circulation of water masses, upwelling nutrients and enhancing productivity [67]
	Bathymetry slope (BS)	Gradient (°) of the sea floor calculated from BATH data at a 0.02° × 0.02° spatial resolution [66]. Ecological importance analogous to BATH [67]
Dynamic variables	Sea-ice concentration (SIC)	Passive microwave estimates of daily sea ice cover (%) extracted at a 25 km x 25 km spatial resolution (the finest resolution available over the whole study period) [68]. Provides insight into open water accessibility or as a resting platform [69]
	Sea surface temperature (SST)	Measured daily in °C at a 0.25° × 0.25° spatial resolution [70]. Reflects the temperature of water masses and fronts, which can influence biological productivity [71]
	Sea surface height (SSH)	Variability of the daily sea surface height (m) at a 0.25° × 0.25° spatial resolution, obtained using E.U, Copernicus Marine Service Information (http://marine.copernicus.eu). Indication of water masses and fronts. Ecological importance analogous to SSH [71]

### Statistical analyses

With the complete final dataset (SSM filtered location estimates integrated with corresponding dive and environmental information) we used the *‘fit_mpm’* function in the ‘*foieGras’* package to fit a random walk with time-varying move persistence model ${(\gamma }_{t})$ [60]. Models were fitted with a single, pooled random variance parameter. This move persistence method captures the autocorrelation in both speed and direction between successive displacements along a horizontal movement trajectory. The time-varying persistence parameter (${\gamma }_{t})$ in horizontal movements is provided on a continuous scale from 0 (low autocorrelation and move persistence indicative of low speed and directionality [residency]) through to 1 (high move persistence indicative of high speed and linear directionality [directed travel]) [36].

To make inferences about how movement persistence (${\gamma }_{t})$ varied in relation to diving effort and environmental features during the chick-rearing period, we also used move persistence mixed effects models [*'mpmm'* function, *'mpmm'* package; 72] which model ${\gamma }_{t}$ as a linear function of environmental predictors measured at each location or time. With this approach, the random walk on logit(${\gamma }_{t}$) employed in *fit_mpm* is replaced with a linear regression of covariates on logit(${\gamma }_{t}$) [36]. Each model was fitted with sex as a fixed factor and individual penguin ID as a random factor. We fitted separate models to guard and crèche foraging trips. For each behavioural and environmental predictor, we configured random intercept only models and random intercept and slope models, the latter allowing for these relationships to vary among individuals.

To build more complex behavioural-environment models, we first inspected correlation coefficients of predictor variables to determine collinearity. Behavioural predictors were generally more highly correlated than environmental predictors (Fig. S2) We therefore adopted a step-forward approach (starting from the null model) based upon AIC where the best supported behavioural and two best supported environmental models were carried forward into more complex behavioural-environment model configurations. Adopting a step-backward approach from a full model including all possible covariates was not possible given the computationally intensive approach, and configuring complex random effects structures can also result in convergence issues. This process was considered pragmatic and sufficient to explore movement-behaviour-environment relationships.

The performance of our final models was assessed using a “leave-one-out” cross-validation method, where we iteratively excluded one individual penguin ID, re-ran the model with the remaining data and examined coefficient estimates in relation to the full model output. All diving metrics and BATH were log-transformed except SIC which was logit transformed. All behavioural and environmental predictors (except SIC) were scaled and centred to aid model convergence. Model terms were considered significant at *p*-value < 0.05.

## Results

### Trip characteristics

**Fig. 1:**
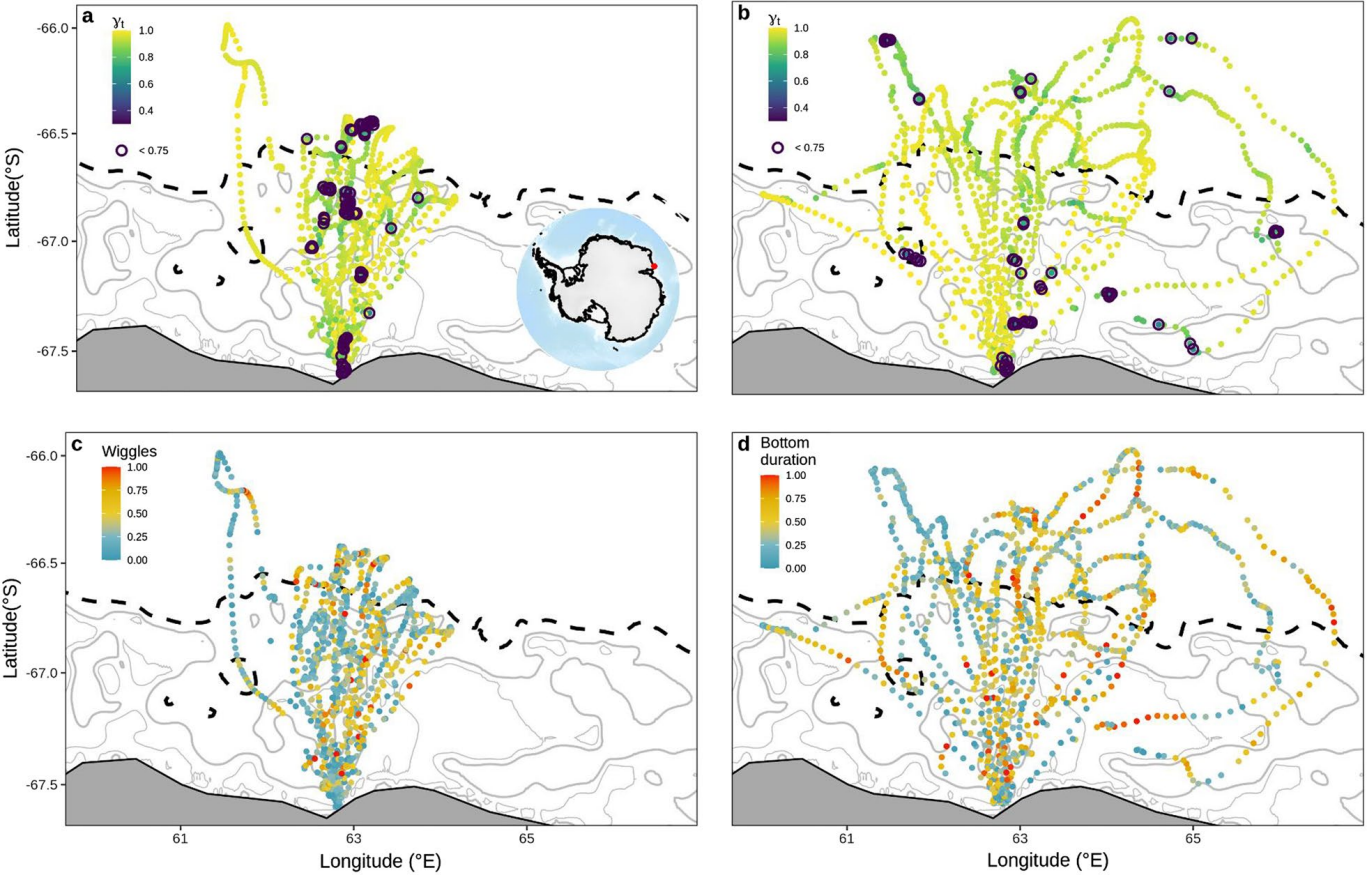
Map of SSM-filtered location estimates for chick-rearing Adélie penguins (n = 23 individuals on n = 27 foraging trips) at Béchervaise Island*.* Top panels illustrate move persistence values ${(\gamma }_{t})$, for guard (**a**) and creche (**b**), where darker colours indicate lower autocorrelation in speed and directionality along animal movement trajectories (see Methods for details). Move persistence values < 0.75 are emphasised in dark blue open circles. Bottom panels show location estimates coloured by wiggles during guard (**c**) and bottom duration during crèche (**d**), which were selected as the best behavioural predictors in move persistence mixed effects models (see Results for details). To standardise across individuals, wiggles and bottom duration are presented relative to the maximum value recorded for each individual foraging trip. Bathymetric contours are displayed at 100 m intervals. Major bathymetric features (shelf break and other bathymetric features > 1000 m), are illustrated by black dashed lines. Major land features are shown in grey. Inset panel in (**a**) shows the study region (red circle) in East Antarctica

Across 23 individuals and 27 foraging trips spanning 6 breeding seasons, a total of 38,845 dives were recorded along the 2220 locations at sea (Table 1). Between chick-rearing stages, there were clear differences in penguin foraging range (Table 3). During guard, penguins rarely travelled beyond the shelf break. However, during crèche, individuals expanded their foraging distribution, ranging farther east and west and foraging north of the shelf break (Fig. 1). Among all foraging trips, the mean foraging trip duration was 98 and 114 h for guard and crèche respectively (Table 3).

**Table 3:** Summaries of chick-rearing Adélie penguin activity at Béchervaise Island (n = 23 individuals, n = 27 foraging trips)

Number of birds = 23	Guard		Crèche	
	(*n* = 14 trips)		(*n* = 13 trips)	
Trip characteristics	Mean (CI)	Range	Mean (CI)	Range
**Foraging trip**				
Duration (h)	98 (62–125)	77–181	114 (57–162)	73–208
Maximum distance from colony (km)	222 (178–249)	199–306	242 (178–283)	203–329
**Move persistence**				
${\gamma }_{t}$	0.89 (0.66–1.00)	0.62–0.99	0.91 (0.71–1.00)	0.32–0.99
**Dive data**				
Depth (m)	265 (30–796)	5–1575	299 (40–836)	5–1879
Bottom duration (s)	506 (99–1164)	19–2259	559 (103–1327)	19–2247
# Wiggles	61 (9–160)	5–568	96 (16–241)	5–628
ACPUE	0.12 (0.03–0.25)	0.07–0.47	0.17 (0.08–0.26)	0.09–0.64

Values represent the geometric mean (95% confidence interval) and range for guard and crèche. Maximum distance from colony (great circle distance) is calculated using the maximum distance travelled for each individual foraging trip. Move persistence and dive data values are calculated across each hour of the foraging trip. See Methods for definitions of move persistence ${(\gamma }_{t})$ and dive metrics

### Move persistence and dive behaviour

The move persistence behavioural index amongst Adélie penguins ranged between 0.3–1 (Table 3; Fig. 1; Additional file 1: Fig. S3). Generally Adélie movement trajectories were dominated by higher move persistence (average ${\gamma }_{t}$ values of 0.89 and 0.91 during guard and crèche, respectively; Table 3). Overall, just 8% of location estimates (n = 159) from 13 trips (48% of the total) recorded move persistence values below 0.75. During guard and crèche, some movement trajectories featured patches of lower move persistence throughout trip loops (Fig. 1a, b). For both chick-rearing stages, dive activity was spread throughout movement trajectories, and areas of pronounced effort did not clearly correspond with particular spatial areas (Fig. 1c, d); relationships with move persistence are quantitatively examined below.

### Horizontal-vertical movement relationships

#### Guard

**Fig. 2:**
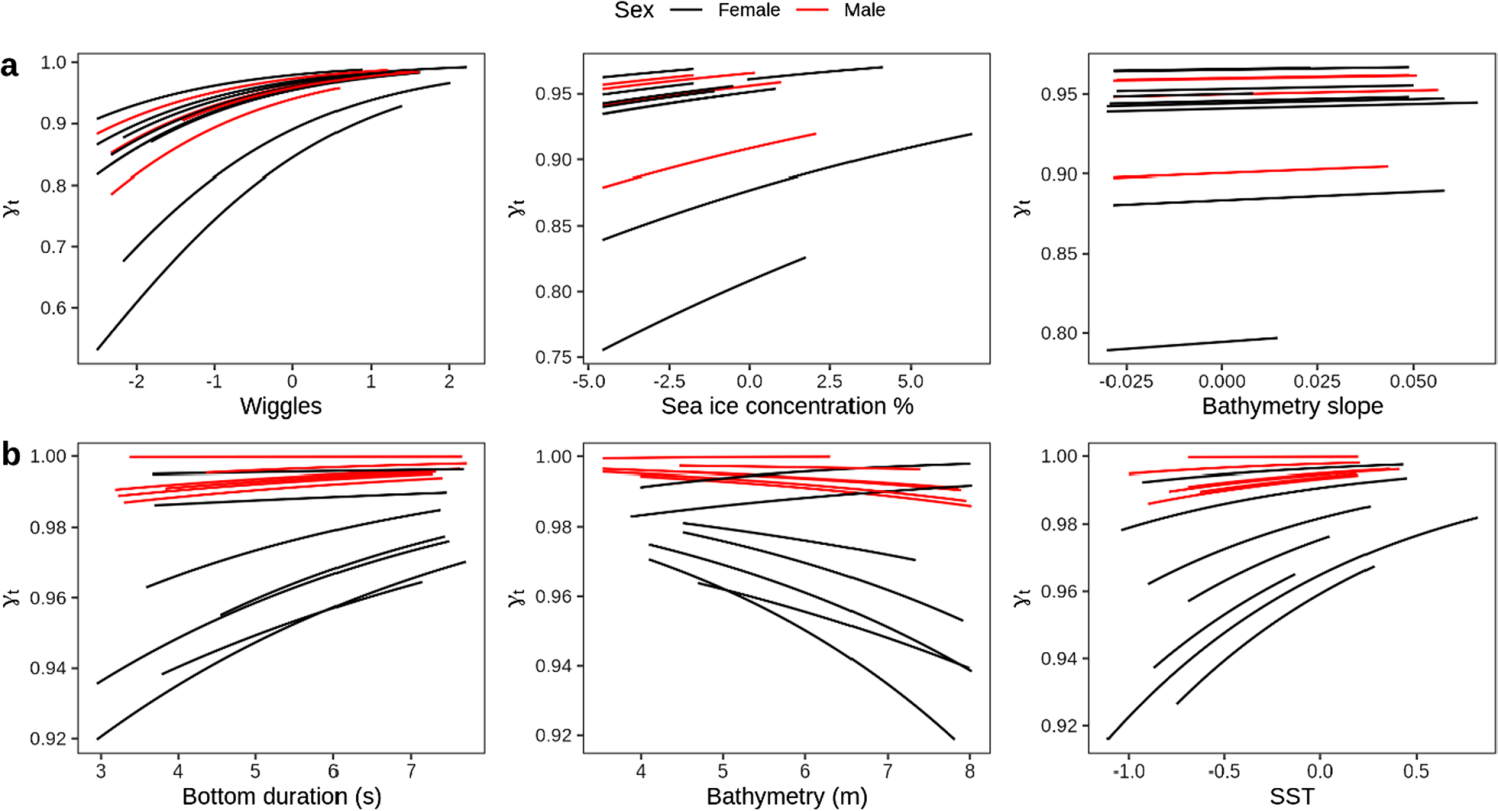
Results of best supported behavioural-environmental move persistence mixed effects model configurations for individual **a** guard and **b** crèche trips. For both stages, move persistence is modelled in relation to the best supported behavioural predictor and the two best environmental predictors (see Methods for further detail and Tables S2 and S3 for model results). Plots are coloured by sex (females: black; males: red) and all individuals are displayed relative to their x-axis range. Wiggles, bottom duration, and BATH are natural log transformed, and SIC logit transformed. All behavioural and environmental predictors, except SIC, are also scaled and centred, as described in Methods. Full model results are provided in Table 4

During guard, the number of wiggles during a dive was the best supported behavioural predictor (random intercept only model) for changes in Adélie penguin move persistence (Table S2). Dive depth (random intercept and slope model) provided the second best-ranked behavioural predictor. Bathymetry slope (BS) followed by sea-ice concentration (SIC) (both random intercept only models) were the two best supported environmental predictors for changes in Adélie penguin move persistence during guard (Additional file 1: Table S2). The final model configuration incorporating the best supported behavioural and two best environment predictors showed Adélie penguins consistently increased move persistence in association with increasing SIC and BS, and most notably in association with increased underwater wiggle activity. Move persistence did not differ between sex during guard (Fig. 2a; Table 4). Supporting these findings, when configured with dive depth (the second-best behavioural predictor) rather than wiggles, similar behavioural relationships were reported with lower move persistence associated with reduced vertical dive effort (estimated depth coefficient: 0.56 ± 0.05, z-value = 10.90, *p* value < 0.0001). Cross-validation of the final guard model showed the estimated coefficients were consistent with those from the full model in at least 91% of iterations (Table 4).

**Table 4:** Results of move persistence mixed effects models for behavioural-environmental model configurations

Model formula	AIC	LogLik	Fixed effects	Coefficients					
				Est	SE	z-value	*p* value	CV QR	Est trend (%)
**Guard**									
~ Wiggles + SIC + BS + Sex + (1| id)	− 12,464	6241	Intercept	3.52	0.20	17.71	** < 0.0001**	3.05–4.17	100
			Wiggles	0.65	0.01	6.84	** < 0.0001**	0.59–0.93	100
			SIC	0.07	0.01	2.84	** < 0.001**	– 0.03–0.26	91
			BS	1.06	0.01	12,138.9	** < 0.0001**	0.89–1.79	100
			Sex	0.20	0.20	0.89	0.38	– 0.21–1.29	82
**Crèche**									
~ Bottom duration + BATH + SST + Sex + (Bottom duration + BATH | id)	− 14,245	7132	Intercept	3.67	0.10	108.23	** < 0.0001**	1.69–4.25	100
			Bottom duration	0.20	0.01	5.51	** < 0.0001**	– 0.22–0.81	88
			BATH	− 0.20	0.60	− 3.85	** < 0.0001**	– 0.54–0.62	38
			SST	0.83	0.20	52.01	** < 0.0001**	– 0.24–1.68	88
			Sex	1.75	0.1	150.19	** < 0.001**	1.32–2.12	100

Move persistence is modelled in relation to the best supported behavioural and environmental predictors separately for guard and crèche, as determined through AIC ranking (see Methods and Tables S2 and S3 for details). Estimated means ± SE are presented for wiggles, bottom duration and bathymetry on the natural log scale and logit scale for SIC. All behavioural and environmental predictors, except for SIC, were centred and scaled. Females are the reference level for the categorical Sex term. Model terms with a significant *p*-value at the 0.05 level are highlighted in bold text. 95% quantile range (QR) of coefficients from the “leave-one-out” cross validation (CV) are also presented alongside the percentage of CV model fits in which the estimated coefficients show a sign consistent with the final full model coefficients (refer to *Methods* for details)

#### Creche

The best supported behavioural predictor of Adélie penguin move persistence during crèche was bottom duration, followed by dive depth (both random intercept and slope models). The best available environmental predictors during crèche were bathymetry (random intercept and slope model) and SST (random intercept only model) (Table S3). The final crèche model configuration incorporating the best supported behavioural and environment predictors showed movement became slower and more resident (lower move persistence) as penguins reduced the amount time in the bottom phase and entered a with cooler SST and deeper bathymetry. The relationship between move persistence and bathymetry was variable amongst individuals, with three penguins displaying the opposite trend, i.e., decreasing speed and directionality over shallower bathymetry. Male movement trajectories were generally more persistent than females during crèche (Fig. 2b; Table 4). When configured with dive depth (second best-ranked behavioural predictor) rather than bottom duration, the behavioural relationship with move persistence inverted, with penguins reducing speed and directionality with increased vertical dive movement (estimated depth coefficient: − 0.24 ± 0.09, z-value = − 2.60, *p* value < 0.001). Our cross-validation procedure produced coefficients consistent with those from the final crèche model in at least 88% of iterations (Table 4), an exception being the much less reliable estimation for the bathymetry relationship (38%), likely due to the individual variability described above.

## Discussion

We integrated spatial location, dive and environmental data for chick-rearing Adélie penguins at Béchervaise Island to test the relationship between a putative index of foraging in the horizontal dimension, vertical dive effort and environmental variables. Our findings show lower move persistence in horizontal movement trajectories did not correspond with intensive periods of dive activity. This contradicts horizontal-vertical movement expectations derived from OFT, which implies ARS strategies in the horizontal dimension should correspond with pronounced dive effort. Our results therefore challenge how important foraging habitat might be identified for this marine predator. This finding contributes to the growing body of literature demonstrating straightforward interpretations of horizontal movements, based on OFT, do not always match foraging effort in the vertical dimension and likely oversimplifies three-dimensional habitat use. Our movement-environment results also highlight the strong seasonal aspect to foraging habitat for chick-rearing Adélie penguins at this colony.

### Instrument effects

The effects of externally attached data loggers must also be considered as a potential source of bias in this study. The additional weight and hydrodynamic drag of external attached bio-logging devices can have a negative impact on penguin movements [73]. Therefore, two standard PTT and TDR devices deployed simultaneously may have had an influence on Adélie penguin horizontal and vertical movement in this study. While we cannot discount these potential effects to fine-scale movement behaviour (i.e. foraging range and dive performance), we believe the broad-scale horizontal-vertical movement strategies revealed by this study would not be significantly affected.

### Underwater foraging strategies

Integrating dive and location information can provide a spatial and temporal context for marine predator foraging effort [6, 7, 67]. We found wiggles and bottom duration, during guard and crèche respectively, were the best predictors of Adélie penguin move persistence at Béchervaise Island. While depth was the second-best ranked behavioural predictor during both stages, the relationship with move persistence differed. These behavioural results demonstrate variation in underwater foraging strategies across chick-rearing stages, likely driven by seasonal variation in environmental conditions and associated prey dynamics [43, 74, 75]. Wiggles and bottom duration, which are both considered reliable correlates of prey ingestion [9, 76], became more pronounced as penguins travelled with a higher speed and linear directionality in the horizontal dimension. This finding indicates chick-rearing Adélie penguins at Béchervaise Island do not adopt a horizontal ARS foraging strategy where foraging effort is concentrated in spatially discrete patches. As recorded for other marine predators, such as southern bluefin tuna (*Thunnus maccoyiii*) [77, 78], penguins from this colony appear to forage less in areas where they spend the most time. It seems likely that more residential movement trajectories represent lower intensity foraging or resting behaviour at sea [51].

It is important to recognise our analysis removed dives which were < 3 m to account for TDR surface noise. In doing so, it is possible this may have excluded certain shallow water transiting (i.e. surface porpoising) and surface foraging activities (i.e. under ice) [9]. This may potentially act as a source of bias in our horizontal-vertical movement analysis. Nevertheless, our horizontal-vertical behavioural findings are comparable with other Adélie penguin studies [48, 79], reinforcing the idea that this marine predator’s foraging behaviour should not necessarily be viewed through a horizontal ARS lens.

Adélie penguin movement trajectories at Béchervaise Island were characterised by generally high speed and linear directionality (high move persistence). While move persistence did vary over the course of trips for most individuals, the magnitude of these changes were smaller than documented for other wider ranging Southern Ocean marine predators [72]. As a species which perform relatively short and intense foraging trips during the chick-rearing period, it is perhaps unsurprising penguins displayed consistently high move persistence. Based on the assumption that foraging occurs during times when penguins display more wiggles and longer at dive bottom depths, this behaviour suggests Adélie penguins at this colony forage continuously and intensively over their horizontal movements at sea. Our findings do not support a conceptual model where central-place marine predators “shuttle” commute from a breeding to a feeding location, but rather forage more diffusively within prey patches along their pathways at sea [41, 67, 80].

Our behavioural results might indicate chick-rearing penguins at this colony forage within a region of generally high prey availability rather than encountering concentrated patches of prey across the seascape. For this breeding colony, diving behaviour is characterised by a high degree of bout activity throughout guard and crèche [43]. As reported for Adélie penguins in the Ross Sea, horizontal and vertical ocean transport may continually replenish the local prey-field, providing high prey-availability [48]. If this is the case for foraging penguins at Béchervaise Island, all dives might essentially be performed within an accessible prey-field [6], with low cost associated with inter-patch movement. However, observed inter-annual fluctuations in breeding success and meal mass [75, 81], coupled with potential nearshore prey-depletion during crèche [43], likely confound this ecosystem hypothesis. Further information on the inter-annual variation in the regional prey-field, in addition to the amount and type of prey consumed, are needed to understand how horizontal-vertical movement relationships directly link to prey consumption.

Alternatively, our behavioural results might also be explained by the impracticability of optimal foraging expectations for chick-rearing Adélie penguins who are under intense energetic pressures. During this time, breeding Adélie penguins must acquire enough energy to provision chicks and meet self-maintenance requirements [38]. These intrinsic pressures, coupled with the reality that penguins must forage in an unpredictable heterogenous three-dimensional environmental could mean ARS behaviours are not the most optimal strategy [48], and extensive search behaviour might be more efficient [82, 83]. Within an unpredictable marine environment, travelling past prey capture opportunities with the expectation of encountering richer prey aggregations could result in low foraging success, which in turn, may have profound implications for survival and breeding success. Instead, feeding continuously and opportunistically during foraging trips may be an advantageous alternative [48, 77].

### Movement response to environment

We found SIC affected Adélie penguin movement trajectories at Béchervaise Island during guard, however the nature of this movement-environment relationship was unexpected. At this colony, increased SIC early in the chick-rearing period can restrict access to foraging grounds (Fig. S4). Generally, there is extensive fast-ice adjacent to the colony during the guard stage [84]. We anticipated these environmental conditions might have an influence on our move persistence analysis, with penguins forced to move slowly through areas of dense pack-ice, creating potentially spurious readings of ARS behaviour in movement trajectories [20, 85]. In both marine and terrestrial predators, residency in movement trajectories have been attributed to landscape features (e.g. rough terrain) or other factors (e.g. social behaviour), unrelated to foraging [86–88]. Therefore, it was somewhat surprising that persistent and directed movement behaviour for Adélie penguins at this colony corresponded with high SIC. With increased move persistence associated with pronounced diving activity, as indicated by our behavioural results, it seems penguins at this colony increase underwater foraging activity when encountering areas of high SIC during guard. Our results may be explained by considering ideal foraging habitat for Adélie penguins during guard. Adélie penguins forage in close association with the sea ice during their breeding cycle, preferentially targeting diffuse sea ice concentrations in the marginal ice zones [49]. Within these sea-ice environments, penguins target under-ice dwelling prey items, such as fish and krill [9]. Despite high SIC (as inferred from low resolution satellite data), cracks and small openings in the ice may facilitate access to productive under-ice foraging grounds, enabling penguins to dive repeatedly and move efficiently through the sea-ice environment [51]. Further investigation of Adélie penguin movements at a finer SIC resolution is needed to validate our interpretations of guard foraging for the Béchervaise Island colony (Additional file 1: Fig. S5). This may be feasible for more recent tagging studies where higher resolution sea ice data are available.

The slope of the seafloor also had a strong influence on move persistence during guard. Move persistence was greatest in areas with a relatively steep bathymetry slope. Bathymetric gradients can aggregate prey items through upwelling nutrient-rich waters [67, 89], and have previously been identified as an important environmental feature for Southern Ocean predators foraging in the inner shelf waters of the western Antarctic Peninsula [90]. Adélie penguins and other penguin species can forage in association with a range of bathymetric features, such as seamounts and submarine canyons, due to their high predictability as foraging hotspots [39, 67]. During guard, Adélie penguins at Béchervaise Island frequently forage in association with a relatively localised 200–500 m deep submarine canyon [39]. Together with high SIC, the bathymetry slope can enhance nutrient and prey concentrations [90, 91], providing an important foraging habitat during guard.

Foraging movements were influenced by different environmental conditions during crèche. Our results show SIC was not amongst the best predictors of crèche move persistence. As the chick-rearing season progresses from guard to crèche, SIC decreases and the area becomes an ice-free, or combination of fast- and pack-ice environment [39, 40, 55]. Under these late-season conditions SIC can be expected to have less of an influence on foraging behaviour (Additional file 1: Fig. S4). Instead, bathymetry and SST were found to be better predictors of move persistence. Interpreted in conjunction with our behavioural results, shallow and warmer surface waters appear to be important foraging areas during crèche. Similar to bathymetric features, SST can influence food availability by enhancing primary and secondary productivity in pelagic waters [92]. In the Atlantic sector of the Southern Ocean, warmer surface temperatures support high biological productivity, and as a result, are targeted by marine predators, such as King penguins (*Aptenodytes patagonicus*) [93]. At Béchervaise Island, breeding Adélie penguins can spend a longer time away from nests in crèche and forage within a reduced sea-ice environment. These factors allow parents to explore more distant prey-rich locations, travelling further from their guard foraging grounds and likely encountering different oceanographic conditions and bathymetric features [39, 43]. The importance of SST and bathymetry in crèche is probably a reflection of changes in penguin foraging strategies driven by prey response to seasonal variation in environmental conditions [93].

Within the Mawson region, repeat acoustic surveys have demonstrated seasonal variation in prey biomass [94]. These localised changes in prey abundance and distribution likely induce behavioural responses from foraging Adélie penguins [40]. This is supported by the dietary composition of penguins at Béchervaise Island, which can be highly variable between chick-rearing stages and years, fluctuating between krill and fish-dominated [75]. However, our movement-environment inferences must be validated with coincident information of the regional prey-field. Our findings warrant further investigation into how these static and dynamic environmental features influence the prey field and hence penguin foraging behaviour at this colony.

When interpreting our movement-environment results, it is important to consider the issue of scale. Remotely sensed environmental data can provide valuable ecological insight into the links between movement behaviour and environment features [95]. However, dynamic oceanographic processes are complex and occur over fine spatial and temporal scales [96]. It is possible environmental features influence Adélie penguin move persistence at a spatial and temporal scale we were unable to resolve using relatively coarse remotely sensed data. Furthermore, in this region, the dynamic environmental variables examined can be highly variable between years [40]. This may have a profound influence on spatial distribution and availability of prey, potentially creating variation in behavioural strategies of Adélie penguins between years [9]. Evidently, there is considerable scope for further exploring the environmental drivers of Adélie penguin movement behaviour at this colony.

### Sex differences in movement strategies

We found move persistence did not differ between sexes in guard but was significantly higher for males in crèche. Sex-specific foraging strategies during the chick-rearing period have been relatively well-documented for Adélie penguins at this colony [43, 97, 98]. The lack of sex differences in move persistence parameters may be due to the immense chick-provisioning pressures in guard. It is possible fast, linear and directed movement provide the most optimal foraging strategy for both sexes during this period, even while potentially targeting different habitats and resources [75, 98]. However, it is also plausible our results are biased by the low data available for male trips during guard (Additional file 1: Table S1). In crèche when parents can spend a longer time at sea foraging and access a broader range of habitats, it is plausible there is greater scope and flexibility to optimise movement strategies according to the distribution and aggregation of preferred prey types.

### Modelling perspectives

It is important to consider the limitations and caveats associated with our modelling approach. State-space movement models discriminating discrete movement states have demonstrated their utility for wide-ranging, deep diving marine predators (e.g., seals) and pulse-travel foragers that cannot travel and forage simultaneously (e.g., flying seabirds) [99]. Incorporating time-varying parameters into movement models is a promising approach, which has yielded a more nuanced understanding of the foraging movements of wide-ranging taxa than widely-used two-state switching models [35, 36]. However, it is possible changes in Adélie penguin move persistence may occur at a finer spatial and temporal scale than we were able to resolve in this study, using relatively coarse resolution PTT data [22, 77, 79]. This location uncertainty may have implications for our move persistence behavioural index, which relies on spatial and temporal autocorrelation in successive displacements [61]. Further investigation of penguin movement relationships using more precise, and high-resolution telemetry devices (e.g., GPS) should help to resolve these finer scale processes. Discrete state-switching models provide an alternate tool for characterising Adélie penguin movement patterns [6, 27], and a comparative approach could be useful to validate our conclusions of foraging behaviour and habitat use at this colony.

Furthermore, recent advances in bio-logging technology, such as accelerometers [100], magnetometers [101] and animal-borne video cameras [9], can be used to complement horizontal and vertical movement information gathered from classic telemetry devices. This may provide a spatial and temporal context to actual prey ingestion and feeding events, yielding a more robust understanding of foraging behaviour and horizontal and vertical habitat use.

## Conclusions

By integrating spatial location with dive data and environment information, we provide valuable insight into the horizontal-vertical movements and at-sea foraging behaviour of Adélie penguins. Movement trajectories varied in response to different environmental conditions during guard and crèche, highlighting seasonal variation in habitat use for this colony. Our results reveal a disconnect between putative foraging areas in the horizontal dimension and foraging effort in the vertical dimension for Adélie penguins at Béchervaise Island. Dive behaviour was most pronounced during times of high move persistence, suggesting slower and more resident horizontal movements likely infer resting rather than intensive foraging behaviour. This challenges traditional horizonal-vertical movement expectations for this important marine predator. In remote marine ecosystems where indirect measures are relied upon to guide our understanding of animal foraging, it is imperative ecological inferences are grounded in appropriate theoretical frameworks. This is critical to build our understanding of habitat usage and foraging hotspots across taxa, and ultimately inform ecosystem-level spatial conservation and management.

## Supplementary Information

**Additional file 1**. **Table S1**. Number of male and female Adélie penguins from Béchervaise Island co-tagged with PTT and TDR devices over the 6 breeding seasons examined. **Table S2**. Full model results from move persistence mixed effects models during guard, incorporating relationships with behavioural and environmental predictors. **Table S3**. Full model results from move persistence mixed effects models during crèche, incorporating relationships with behavioural and environmental predictors. **Fig. S1**. Example time-series of move persistence from Adélie penguin foraging trips at irregular (fitted) and regular (1, 2 and 3-hour) time steps. **Fig. S2**. Correlations between predictor variables used to inform final model configurations. **Fig. S3**. Time-series of move persistence from Adélie penguin foraging trips during (**a**) guard and (**b**) crèche, co-plotted with wiggles and bottom duration. **Fig. S4**. Histogram of sea ice concentration chick-rearing Adélie penguins at Béchervaise Island encountered during foraging trips over the study period. **Fig. S5**. Map of SSM-filtered Adélie penguin tracks at Béchervaise Island overlaid with sea-ice cover at 25km and 6.25km resolution, for 4 individuals.

## Data Availability

The dataset used and analysed during the current study are available from the corresponding author on reasonable request and with permission of Louise Emmerson.

## Abbreviations

OFT: Optimal foraging theory
ARS: Area-restricted search
SSM: State-space model
CCAMLR: Commission for the Conservation of Marine Living Resources
CEMP: CCAMLR Ecosystem Monitoring Program
PTT: Platform terminal transmitter
TDR: Time-depth recorder
SIC: Sea-ice cover
AIC: Akaike information criterion
MPM: Move persistence model
GPS: Global positioning system
