# Supplementary material for: Horizontal-vertical movement relationships: Adélie penguins forage continuously throughout provisioning trips
Source: Mov Ecol. 2021 Aug 26;9:43. doi: 10.1186/s40462-021-00280-8 (PMC8393751; doi:10.1186/s40462-021-00280-8)
Supplement: Supplementary file 1 — Additional file 1. Table S1. Number of male and female Adélie penguins from Béchervaise Island co-tagged with PTT and TDR devices over the 6 breeding seasons examined. Table S2. Full model results from move persistence mixed effects models during guard, incorporating relationships with behavioural and environmental predictors. Table S3. Full model results from move persistence mixed effects models during crèche, incorporating relationships with behavioural and environmental predictors. Fig. S1. Example time-series of move persistence from Adélie penguin foraging trips at irregular (fitted) and regular (1, 2 and 3-hour) time steps. Fig. S2. Correlations between predictor variables used to inform final model configurations. Fig. S3. Time-series of move persistence from Adélie penguin foraging trips during (a) guard and (b) crèche, co-plotted with wiggles and bottom duration. Fig. S4. Histogram of sea ice concentration chick-rearing Adélie penguins at Béchervaise Island encountered during foraging trips over the study period. Fig. S5. Map of SSM-filtered Adélie penguin tracks at Béchervaise Island overlaid with sea-ice cover at 25km and 6.25km resolution, for 4 individuals. [file 40462_2021_280_MOESM1_ESM.docx]

**Supplementary material**

Table S1. Number of male and female Adélie penguins from Béchervaise Island co-tagged with PTT and TDR devices over the 6 breeding seasons examined. The number of foraging trips per season and chick-rearing period (guard and crèche) are also presented.

|  |  | **Birds** | **Total trips** | **Guard trips** | **Crèche trips** |
| --- | --- | --- | --- | --- | --- |
| **1994-95** | **Female** | 1 | 1 | 1 | 0 |
|  | **Male** | 0 | 0 | 0 | 0 |
|  | **Total** | 1 | 1 | 1 | 0 |
| **1995-96** | **Female** | 0 | 0 | 0 | 0 |
|  | **Male** | 1 | 1 | 1 | 0 |
|  | **Total** | 1 | 1 | 1 | 0 |
| **1998-99** | **Female** | 1 | 3 | 2 | 1 |
|  | **Male** | 1 | 1 | 1 | 0 |
|  | **Total** | 2 | 4 | 3 | 1 |
| **2001-02** | **Female** | 7 | 8 | 5 | 3 |
|  | **Male** | 7 | 7 | 1 | 6 |
|  | **Total** | 14 | 15 | 6 | 9 |
| **2002-03** | **Female** | 3 | 4 | 1 | 3 |
|  | **Male** | 1 | 1 | 1 | 0 |
|  | **Total** | 4 | 5 | 2 | 3 |
| **2003-04** | **Female** | 1 | 1 | 1 | 0 |
|  | **Male** | 0 | 0 | 0 | 0 |
|  | **Total** | 1 | 1 | 1 | 0 |
| **Total** | **Female** | 13 | 17 | 10 | 7 |
|  | **Male** | 10 | 10 | 4 | 6 |
|  | **Total** | 23 | 27 | 14 | 13 |

Table S2. Results from move persistence mixed effects models (mpmm) incorporating relationships with behavioural and environmental predictors. Single-term models are fit to data from 13 penguins during the guard period and evaluated using Akaike information criterion (AIC). The models are ranked according to the best fits (lowest absolute AIC and **Δ**AIC. Best ranked models were carried forward into more complex models combining behavioural and environmental predictors (see *Methods* for further detail).

| **Guard models** | **AIC** | **ΔAIC** | **LogLik** | |
| --- | --- | --- | --- | --- |
| **Behavioural** |  |  |  | |
| ~ Wiggles + Sex + (1 \| id) | -12473 | 0 | 6244 | |
| ~ Depth + Sex + (Depth \| id) | -12469 | 4 | 6238 | |
| ~ Wiggles + Sex + (Wiggles \| id) | -12464 | 9 | 6241 | |
| ~ Bottom Duration + Sex + (1 \| id) | -12460 | 13 | 6237 | |
| ~ Bottom Duration + Sex + (Bottom Duration \| id) | -12457 | 16 | 6238 | |
| ~ ACPUE + Sex + (ACPUE \| id) | -12449 | 24 | 6234 | |
| ~ Depth + Sex + (1 \| id) | -12441 | 32 | 6228 | |
| ~ ACPUE + Sex + (1 \| id) | Did not converge | | | |
| **Environmental** |  |  |  | |
| ~ BS + Sex + (1 \| id) | -12447 | 0 | | 6231 |
| ~ SIC + Sex + (1 \| id) | -12441 | 4 | 6229 | |
| ~ SSH + Sex + (SSH \| id) | -12442 | 5 | 6230 | |
| ~ SST + Sex + (SST \| id) | -12442 | 5 | 6231 | |
| ~ SIC + Sex + (SIC \| id) | -12441 | 6 | 6229 | |
| ~ BATH + Sex + (1 \| id) | -12441 | 6 | 6228 | |
| ~ BS + Sex + (BS \| id) | -12439 | 8 | 6228 | |
| ~ SSH + Sex + (1 \| id) | -12439 | 8 | 6227 | |
| ~ SST + Sex + (1 \| id) | -12433 | 14 | 6223 | |
| ~ BATH + Sex + (BATH \| id) | Did not converge | | | |

Table S3. Results from move persistence mixed effects models (mpmm) incorporating relationships with behavioural and environmental predictors. Single-term models are fit to data from 10 penguins during the crèche period and evaluated using Akaike information criterion (AIC). The models are ranked according to the best fits (lowest absolute AIC and **Δ**AIC. Best ranked models were carried forward into more complex models combining behavioural and environmental predictors (see *Methods* for further detail).

| **Crèche models** | **AIC** | **ΔAIC** | **LogLik** | |  |
| --- | --- | --- | --- | --- | --- |
| **Behavioural** |  |  |  | |  |
| ~ Bottom Duration + Sex + (Bottom Duration \| id) | -14188 | 0 | 7103 | |  |
| ~ Bottom Duration + Sex + (1 \| id) | -14180 | 8 | 7097 | |  |
| ~ Depth + Sex + (Depth \| id) | -14178 | 10 | 7098 | |  |
| ~ ACPUE + Sex + (1 \| id) | -14175 | 13 | 7095 | |  |
| ~ Wiggles + Sex + (1 \| id) | -14168 | 20 | 7091 | |  |
| ~ Wiggles + Sex + (Wiggles \| id) | -14168 | 20 | 7091 | |  |
| ~ Depth + Sex + (1 \| id) | -14138 | 50 | 7076 | |  |
| ~ ACPUE + Sex + (ACPUE \| id) | -14131 | 57 | 7075 | |  |
| **Environmental** |  |  |  | |  |
| BATH + Sex + (BATH \| id) | -14231 | 0 | | 7125 |  |
| SST + Sex + (1 \| id) | -14190 | 41 | 7102 | |  |
| SIC + Sex + (SIC \| id) | -14188 | 43 | 7103 | |  |
| BS + Sex + (1 \| id) | -14141 | 90 | 7078 | |  |
| SSH + Sex + (1 \| id) | -14138 | 93 | 7076 | |  |
| BATH + Sex + (1 \| id) | -14137 | 94 | 7075 | |  |
| BS + Sex + (BS \| id) | -14135 | 96 | 7077 | |  |
| SSH + Sex + (SSH \| id) | -14135 | 96 | 7076 | |  |
| SIC+ Sex + (1 \| id) | Did not converge | | | |  |
| SST + Sex + (SST \| id) | Did not converge | | | |  |


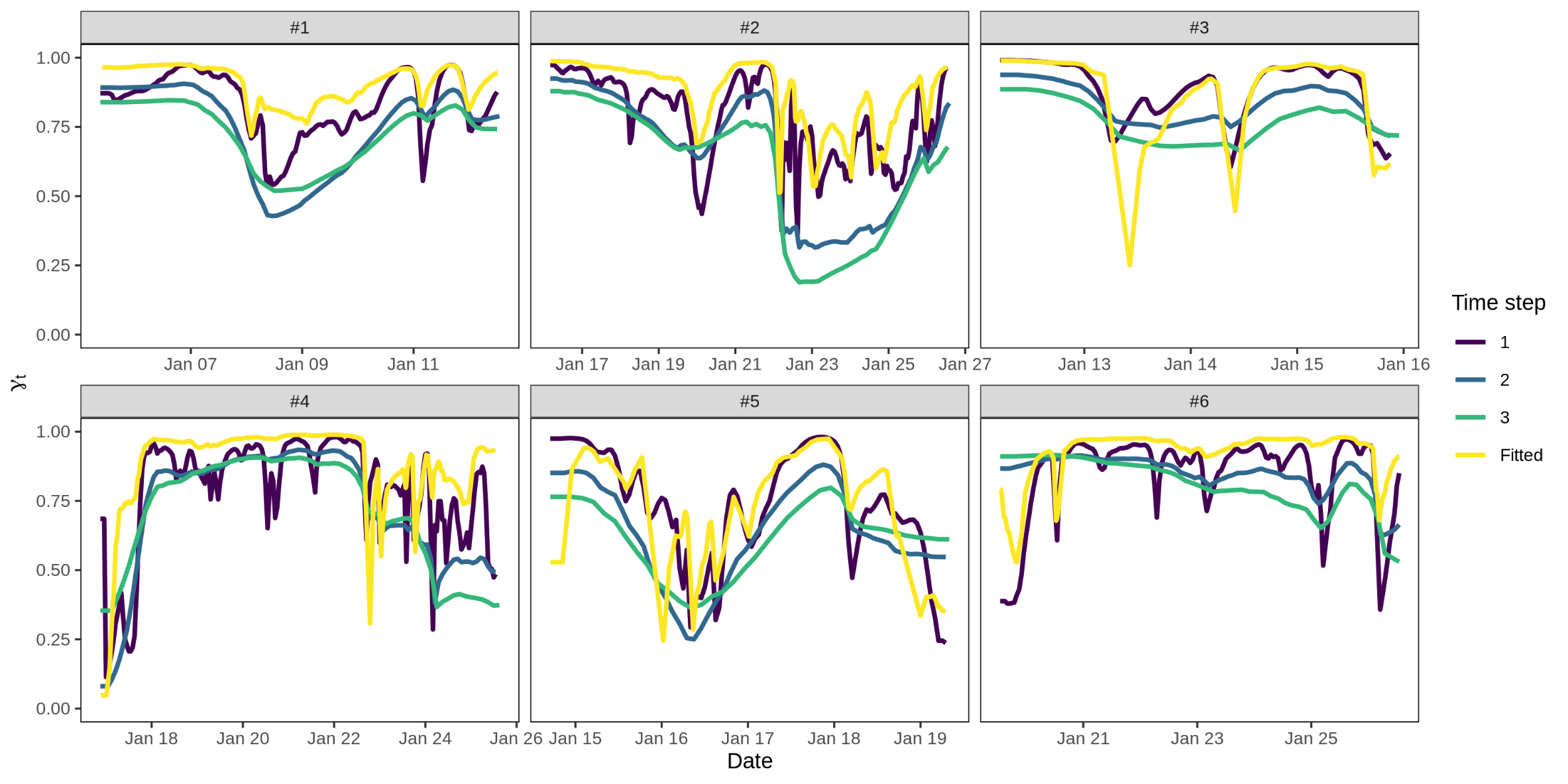


Fig. S1. Example time-series of move persistence from Adélie penguin foraging trips (n = 6) at irregular (fitted) and regular (1, 2 and 3-hour) time steps. Move persistent parameters recorded for 1-hour time steps closely resembled fitted values and was therefore deemed most practical and adequate for our purposes.


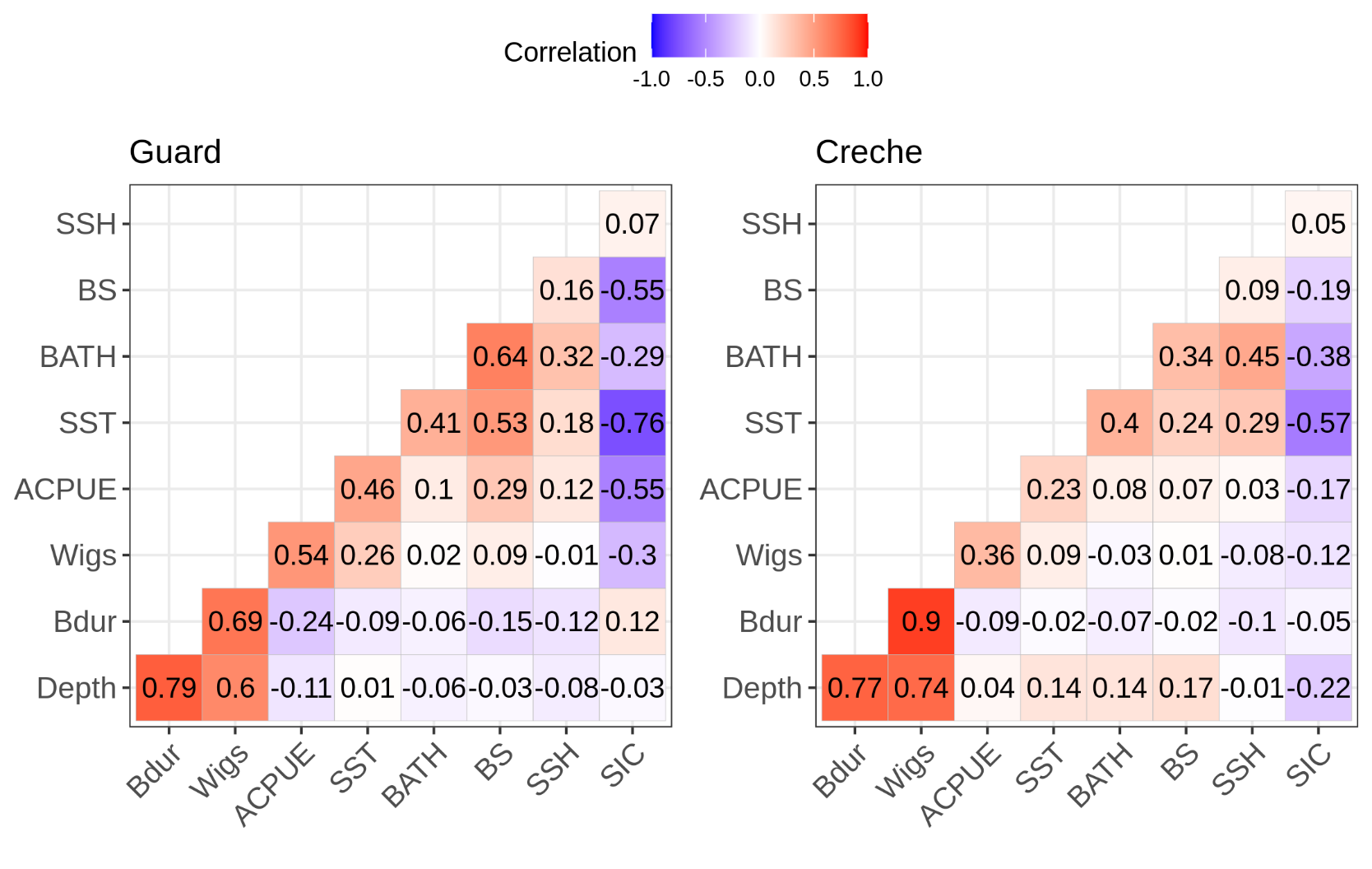


Fig. S2. Correlations between predictor variables used to inform final model configurations. Behavioural and environmental variables are displayed for guard and crèche separately. SSH: Sea surface height, BS: Bathymetry slope, BATH: Bathymetry, SST: Sea surface temperature, SIC: Sea ice concentration, ACPUE: Attempts of catch per unit effort, Wigs: Wiggles, Bdur: Bottom duration, Depth: Depth.


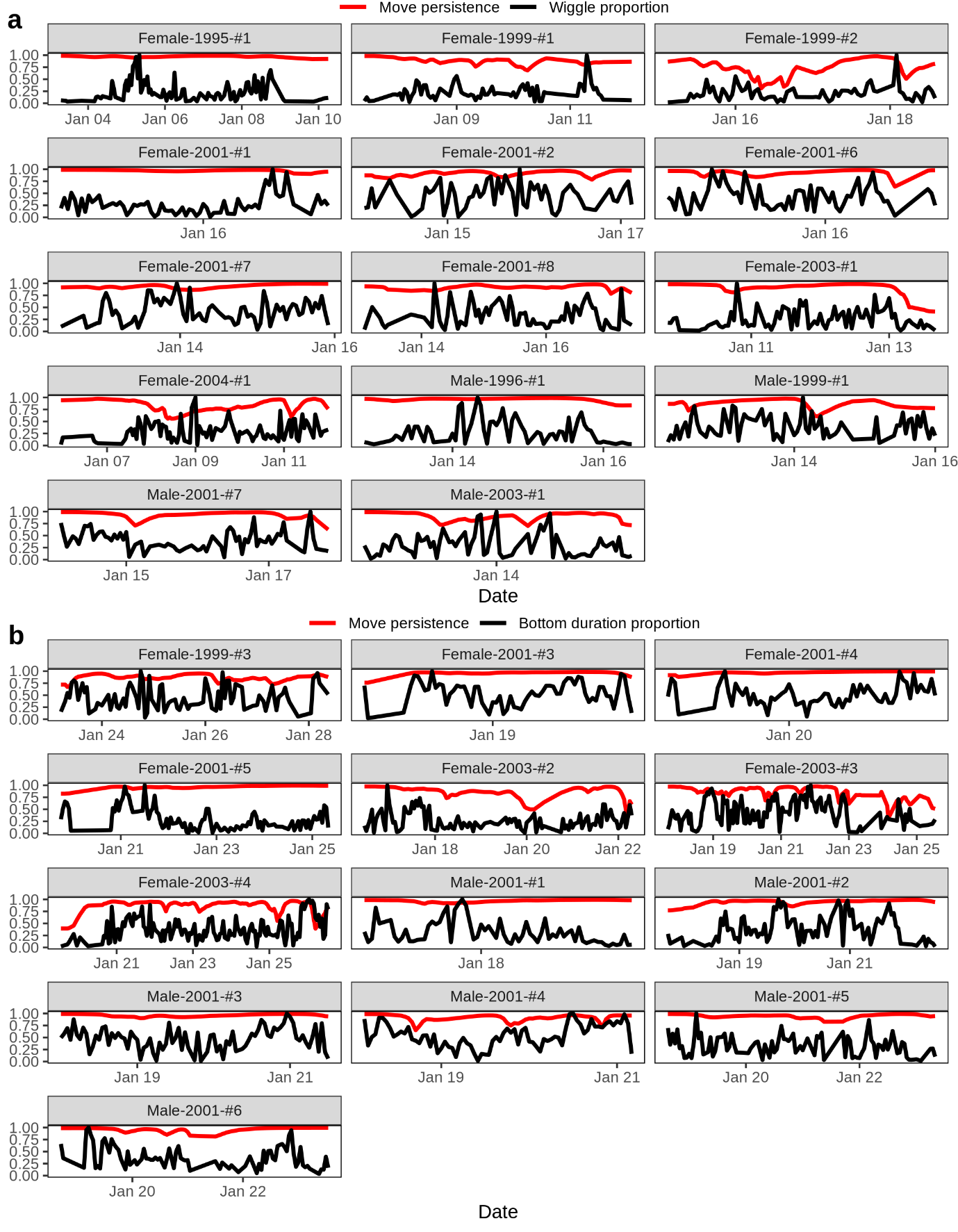
Fig. S3. Time-series of move persistence (red) from Adélie penguin foraging trips (n = 27) during (a) guard and (b) crèche, co-plotted with wiggle and bottom duration, respectively, which were selected as the best behavioural predictors in move persistence mixed effects models. To standardise move persistence and dive activity scales, wiggles and bottom duration data are presented as a proportion relative to the maximum value for each individual foraging trip.


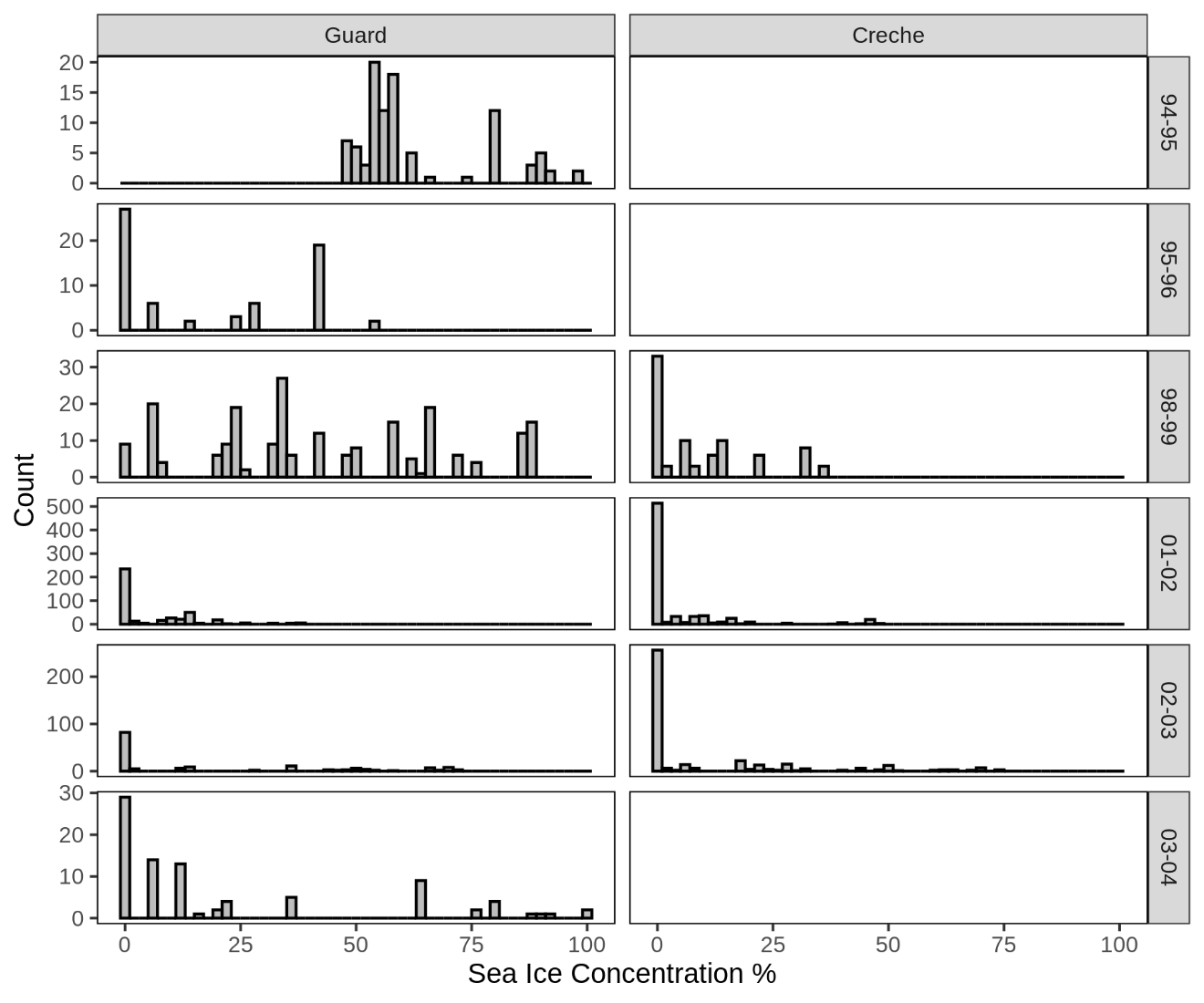


Fig. S4. Histogram of sea ice concentration chick-rearing (guard and crèche) Adélie penguins at Béchervaise Island encountered during foraging trips (n = 27) over the study period from 1994/1995 to 2003/2004.


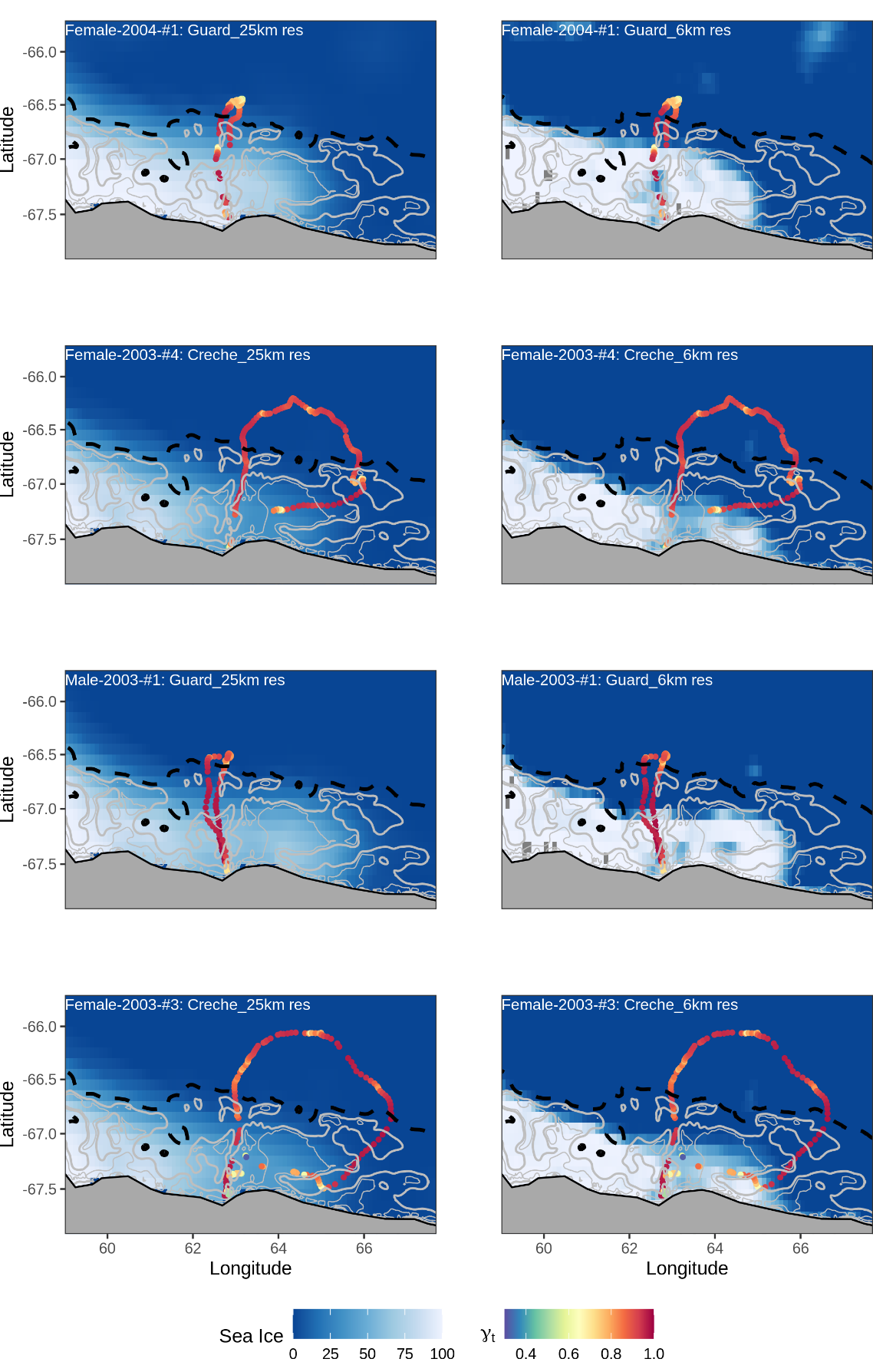


Fig. S5. Map of SSM-filtered Adélie penguin tracks at Béchervaise Island overlaid with sea-ice cover (SIC) for 4 individuals. Left and right-side panels display SIC at a 25km and 6.25km resolution, respectively. Adélie penguin tracks are coloured in accordance with move persistence values derived from the *‘fit_mpm’* function in the ‘*foieGras’* package [60] (see *Methods* for details). Bathymetric contours are displayed at 100m intervals. Major bathymetric features (shelf break and other bathymetric features > 1000m), are illustrated by black dashed lines. Major land features are shown in grey.
